# The Impact of Total Hip Arthroplasty on the Incidence of Hip Fractures in Romania

**DOI:** 10.3390/jcm14134636

**Published:** 2025-06-30

**Authors:** Flaviu Moldovan, Liviu Moldovan

**Affiliations:** 1 Orthopedics—Traumatology Department, Faculty of Medicine, George Emil Palade University of Medicine, Pharmacy, Science, and Technology of Targu Mures, 540142 Targu Mures, Romania; 2 Faculty of Engineering and Information Technology, George Emil Palade University of Medicine, Pharmacy, Science, and Technology of Targu Mures, 540142 Targu Mures, Romania; liviu.moldovan@umfst.ro

**Keywords:** total hip arthroplasty, hip fracture, orthopedics surgery, osteoporosis, public health, epidemiology, implants

## Abstract

**Background/Objectives**: The increase in life expectancy and the share of the elderly population has the effect of increasing the number of osteoporotic hip fractures. At the same time, the number of total hip arthroplasty (THA) interventions is continuously increasing. The objective of this study is to investigate the incidence rates of hip fractures during the period 2008–2019, in Romania, among people who are at least 40 years old, as well as to determine the extent to which the increase in the prevalence of people who have undergone THA has affected the incidence of hip fractures, given that the operated hip no longer presents a risk of fracture. **Methods**: We extracted the information, between 2008 and 2019, from nationwide retrospective studies about the incidence and time trend of hip fractures in Romania. Information on primary THA interventions during the period 2001–2019 was obtained from the Romanian Arthroplasty Register (RAR). We obtained the population size, by gender and age groups, from the reports of the National Institute of Statistics. For the period 2008–2019, we calculated the standardized annual hip fracture incidence rates by sex and by age. Given that each person has two hips at risk of fracture, we calculated hip fracture rates in a scenario without THA interventions. For this, we subtracted 0.5 people from the at-risk population for each prevalent hip prosthesis. Thus, we revealed the effects of decreasing fracture rates due to having hip prostheses. **Results**: From 2008 to 2019, age-standardized incidence rates of hip fractures increased by 10.8% in women, and by 2.8% in men. By excluding hips being replaced with prostheses in the at-risk population, we obtained higher hip fracture incidence rates. These recorded values were considerably higher for the elderly population. The variation in hip fracture rates during the observed period was 10.16% (9.76% in women and 11.68% in men) lower due to the increased prevalence of hip prostheses. **Conclusions**: Although the incidence of hip fractures has continued to rise, the growing number of people who have undergone THA and are living with hip prostheses has helped to blunt this increase.

## 1. Introduction

Osteoporotic hip fractures, also known as fragility fractures, reflect compromised bone strength [1]. They are associated among older adults with serious morbidity, reduced quality of life, disability, and an increased mortality rate [2]. Hip fracture incidence rates show a wide variation in incidence between countries [3]. In recent decades, most advanced countries have seen stagnation or even a decrease in age-adjusted hip fracture incidence rates [4]. However, in Europe, the risk of hip fracture in people after the age of 50 is comparable to the risk of stroke [5].

In the coming decades, the continued increase in life expectancy and population aging will increase the share of people over 60 years [6]. At the same time, the number of hip fractures and the associated societal burden will increase [7]. This requires conducting studies and adopting measures that will generate decreasing hip fracture rates. The causes of variations in hip fracture rates are not well understood, although several factors have been suggested to influence it [8]. However, there is an increase in the number of total hip arthroplasty (THA) interventions and the number of people living with hip prosthesis [9]. Hip osteoarthritis is the dominant indication for THA [10]. This is a chronic and progressive condition that results in progressive loss of cartilage with age [11]. With increasing life expectancy and the proportion of the elderly population, THA rates are expected to continue to increase [9]. At the same time, the average age at which THA is performed is decreasing [12].

In our study, we assumed that the THA-operated hip no longer presents a risk of fracture. Based on this finding, we hypothesized that the increasing proportion of people living with hip prostheses contributes to the decrease in hip fracture rates in Romania.

The objectives of our study are to investigate the incidence rates of hip fractures, during the period 2008–2019, in Romania, among people who are at least 40 years old and to determine the extent to which the increase in the prevalence of people who have undergone THA has affected the incidence of hip fractures.

## 2. Materials and Methods

### 2.1. Study Population and Data Sources

In this modeling study, we used data from the bibliographic references cited in the paper. The data were processed in accordance with the General Data Protection Regulation and did not require ethical approval.

The study covered the Romanian population aged 40 and over, during the period 2008–2019. We extracted the population size by sex and age group, for each calendar year in the studied period, from the reports of the National Institute of Statistics [13,14], Eurostat Population structure [15], and specialized sites regarding the population of Romania [16].

We collected information on patients hospitalized with a diagnosis of hip fracture in specialized medical care in Romania from nationwide retrospective studies [17,18,19]. In these studies, primary data on hip fractures reported by hospitals in Romania were collected. The collection was carried out by diagnostic-related groups (DRGs), at 5-year intervals, for the population stratified into women and men. Precise definitions with diagnostic codes and surgical procedures were used. The information refers to patients admitted to hospitals throughout the country with primary or secondary diagnoses of hip fracture, coded ICD 10 S72.0 (femoral neck).

Information on primary total hip replacement surgeries in Romania during the study period 2008–2019, as well as the previous period 2001–2007, was obtained from the Romanian Arthroplasty Register (RAR) [20]. This register collects individual reports from the 135 orthopedic hospitals that must report on their arthroplasty activity, according to the Ordinance of the Minister of Health no. 1591/1110/2010. Registration in the RAR is based on the informed consent of patients. The RAR database has been compiled since 2001 and is considered a full degree of registration for primary total hip replacements.

### 2.2. Building the Model

We calculated hip fracture incidence rates by sex and by 5-year age groups, from 40 to 85+ years, in three 4-year time periods: 2008–2011, 2012–2015, and 2016–2019. We standardized hip fracture rates by age using the direct method and the 2019 population distribution, separately for women and men. Using these rates, we investigated trends in hip fracture prevalence from 2008 to 2019.

The total number of people with hip replacements in 2019 was estimated by calculating the number of primary replacements for all hip fracture recommendations as well as revision surgeries, performed between 2001 and 2019 in people who were alive and at least 40 years old.

Although the number of people with total hip arthroplasties in 2019 was based on 19 years of reporting to the RAR (2001–2019), data on total hip arthroplasty interventions were only available seven years before 2008. To estimate the total number of hip replacements prevalent in 2008, we used information from the RAR for the period 2001–2007 and evaluated the number of interventions performed after 1990 by following the linear growth trend for the period in which records were made.

To explain the impact of total hip arthroplasty on the incidence of hip fractures in Romania, we were inspired by the NOREPOS model, validated in Norway via the Epidemiologic Osteoporosis Study [21]. For this, we explored the possible impact of changing the prevalence of hip prostheses in the population on the incidence of hip fractures, in a scenario without total hip arthroplasties performed. To estimate the incidence of hip fractures in this hypothetical situation, for each prevalent hip prosthesis we subtracted 0.5 people from the population at risk. This algorithm was used because, when compared with a person with two hips at risk of fracture, people who have undergone THA have only one hip at risk of fracture and consequently a relative risk (RR) of 0.5 for a new hip fracture.

Using the Cox proportional hazards model, we estimated the association between total hip arthropathy and the risk of hip fracture. The time scale was the age reached and the time dependent exposure total hip arthroplasty.

We determined the extent to which the prevalence of hip prostheses in the population contributes to the decrease in the incidence rate of hip fractures with the support of a deterministic model that employs the information between two-time moments, determined by the baseline year (BY) and the end year (EY). This requires statistical data that were employed to calculate a series of variables, as follows:

Statistical data

- Population number (PO), for every gender (G) and age group (AG);
- Observed number of fractures (NO), for every gender (G) and age group (AG);
- Number of hip prostheses (NHP) for every gender (G) and age group (AG).

Variables

- Hip fracture rates in (BY) by sex and age group (r_(BY)_), as follows:
  r_(BY)_ = NO/PO, for every (G) and (AG);(1)

- Considering the population size in the end year PO_(EY)_ and the hip fracture rates in the baseline year (r_(BY)_), we calculated the expected number of hip fractures (NE) in (EY), as follows:
  NE_(EY)_ = PO_(EY)_ × (r_(BY)_), for every (G) and (AG);(2)

- We estimated the number of hip fractures requiring explanation, as the difference between the observed and expected number of hip fractures in (EY), as follows:
  NX^estimated^ = NO_(EY)_ − NE_(EY)_, for every (G) and (AG).(3)

- We calculated the fracture incidence rate in a scenario without THA, as the ratio between the number of observed fractures and the population number from which we extracted 0.5 for each person living with hip prosthesis, as follows:
  rs = NO/(PO − 0.5 × NHP), in (BY) and (EY), for every (G) and (AG);(4)

- The number of hip fractures explained by the influence of hip prostheses factors is calculated as the product between the population size and the difference between the fracture incidence rate in scenario and the fracture incidence rate, as follows:
  NX^explained HP^= PO(_EY)_ × [(rs_(EY)_ − r_(EY)_]. (5)

In our calculation model, we adopted the value RR = 05, but by generalization, for any RR, the number of hip fractures explained by the influence of hip prostheses factors, has the following expression:

(6) $${\mathrm{NX}}^{\mathrm{explained}\mathrm{HP}}={\mathrm{PO}}_{\left(\mathrm{EY}\right)}\times \left[\frac{{\mathrm{NO}}_{\left(\mathrm{EY}\right)}}{{\mathrm{PO}}_{\left(\mathrm{EY}\right)}-\mathrm{RR}\times \mathrm{NHP}}-\frac{{\mathrm{NO}}_{\left(\mathrm{EY}\right)}}{{\mathrm{PO}}_{\left(\mathrm{EY}\right)}}\right],\;\mathrm{in}\;(\mathrm{EY})\;\mathrm{for}\;\mathrm{every}\;(G)\;\mathrm{and}\;(\mathrm{AG}).$$

By summing up the values per “n” studied age groups (AG) the totals per gender (G) is deduced. It can be summed up further for both sexes so that the result indicating the number of hip fractures explained by the influence of hip prostheses factors in the end year is as follows:

(7) $${\mathrm{Total}\mathrm{NX}}^{\mathrm{explained}\mathrm{HP}}=\sum_{G=1}^{2}{\mathrm{NX}}_{\left(G\right)}^{\mathrm{explained}\mathrm{HP}}\sum_{\mathrm{AG}=1}^{n}{\mathrm{NX}}_{\left(\mathrm{AG}\right)}^{\mathrm{explained}\mathrm{HP}}$$

### 2.3. Data Analysis

We calculated 95% uncertainty intervals for the difference between the expected and the observed number of hip fractures that were attributed to hip prostheses, by using the Monte Carlo simulation [22]. We replaced the input parameters with appropriate probability distributions and recalculated the result repeatedly with values randomly sampled from the defined input distributions. Statistical analysis was performed using SPSS–IBM (SPSS, Inc., Chicago, IL, USA) for Windows version 29.0.2 and Excel (Microsoft 365, Albuquerque, NM, USA).

## 3. Results

From 2008 to 2019, the number of hip fractures in women increased by 59.03% (from 7513 to 11,948), and in men it increased by 24.56% (from 4266 to 5314). The age-standardized fracture rate also increased in both women and men during this period (Table 1).

**Figure 1:**
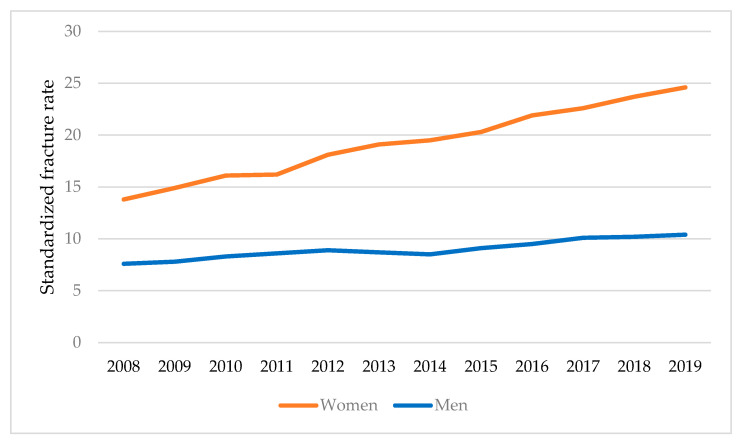
The age-standardized hip fracture rates per 10,000 inhabitants—years in women and men from 2008 to 2019.

In women, the age-standardized rate of hip fractures increased from 13.8 per 10,000 inhabitants in 2008 to 24.6 per 10,000 inhabitants in 2019. In men, the rate of hip fractures per 10,000 increased from 7.6 in 2008 to 10.4 in 2019 (Figure 1).

The ratio of hip fracture rates between women and men also increased from 1.8 in 2008 to 2.4 in 2019.

From the analysis of the number and incidence rate of hip fractures in three four-year periods, 2008–2011, 2012–2015, and 2016–2019, it is found that, for women and men aged 40–64, fracture rates show a slight downward trend in the most recent period. For women and men over 65, fracture rates are continuously increasing (Table 2).

Along with the increasing trend in hip fractures, the number of people living with hip prostheses has also increased (Table 3). In each of the three age groups, the increase between 2008 and 2019 is approximately 2.2 times in women and 2.4 times in men. If, in 2008, a total of 11,779 fractures were observed, in 2019 their number increased to 16,932.

In 2019 the observed number of fractures was 16,932 and the expected number of fractures was 11,675 (Table 4). The number of fractures requiring explanation is 5257 fractures. The number of hip fractures explained by the influence of hip prostheses factors is 536 fractures, i.e., 10.16% (9.76% in women and 11.68% in men).

**Figure 2:**
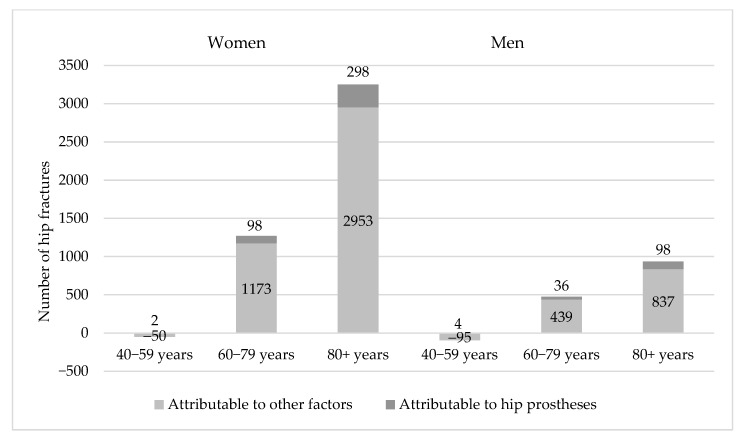
The total heights of the columns represent the difference between the observed and expected number of hip fractures in three age groups in women and men in 2019 given unchanged hip fracture rates since 2008. These are composed of two segments of columns—the top dark parts, which are attributable to the increased prevalence of hip prostheses in the interval 2008–2019 and the bright parts at the base, which are attributable to other factors.

Figure 2 shows the difference between the observed and expected number of hip fractures in 2019.

Differences attributable to the increased prevalence of hip prostheses from 2008–2019 are represented by the dark parts of the columns. The bright parts of the columns represent differences attributable to other factors like variation of body mass index, intensity of physical activities, prevalence of smoking, prevalence of type 2 diabetes, uptake of osteoporosis medication etc. [23].

In the sensitivity analysis assumptions, for a 20% variation in either direction of the prevalence estimate of people living with hip prosthesis in 2008, the uncertainty range increases from 17–18% to only 16–19%.

In this model, for people who have a total hip replacement, we considered RR = 0.5 for a subsequent hip fracture. This assumption was validated in practice through a 17-year observational study [21]. Thus, compared with individuals who did not undergo THA, the relative risk of hip fracture in individuals who had a total hip replacement was RR = 0.5 (95% CI confidence interval, 0.48–0.52).

## 4. Discussion

The analysis of hip fracture epidemiology in Romania between 2008 and 2019 indicates a 46.54% increase in the total number of fractures [17]. However, in the 5-year age groups studied, for people aged 40–64, hip fracture rates show a slight downward trend, while for those over 65, fracture rates are continuously increasing. This increase was slightly greater in men than in women.

Unlike the findings of our study, in most Western countries hip fracture rates have stabilized or started to decrease in recent decades [24]. The effects are due to the stabilization of the urban population, changes in bone mineral density and body mass index, the diagnosis of diabetes [25], the use of osteoporosis medications [26], the use of multivitamins [27], and lifestyle interventions [28] such as improving nutritional status, preventing falls, quitting smoking, and practicing physical exercise.

In this research, we created a new study model, through which we investigated whether the use of total hip prostheses can contribute to decreasing hip fracture rates. We analyzed the hypothetical situation in which the at-risk population would not have undergone total hip arthroplasty interventions. We found that the incidence rates of fractures for this population would have been higher, compared with the observed hip fracture rates. The increase in fracturing frequency would have been greater for people over 80 years of age. In agreement with the study conducted by Moldovan et al. [23], the results of our study indicate that the hip fracture rate from 2008 to 2019 is 10.16% lower due to the increase in the number of people living with hip prostheses.

Although our data are based on national coding systems and do not include specific information on injury mechanisms or detailed anatomical fracture classifications, the analyzed cases predominantly reflect osteoporotic femoral neck fractures (ICD-10 S72.0). These are most often the result of low-energy trauma, such as falls from standing height, particularly among elderly individuals. This mechanism is typical of fragility fractures and is consistent with global trends in osteoporotic hip fractures. However, in contrast to several Western countries that have shown a stabilization or decline in fracture rates due to preventive strategies, Romania continues to report rising incidence rates. This may reflect the limited national implementation of coordinated osteoporosis management, fall-prevention measures, or lifestyle interventions.

Furthermore, while this study focuses on the beneficial impact of total hip arthroplasty (THA) in reducing the risk of hip fracture through the elimination of fracture risk in the operated hip, it is important to consider potential challenges posed by the widespread use of prostheses. THA alters hip biomechanics and gait, which may in some individuals shift mechanical stress toward the contralateral hip. Although our model assumes a relative risk (RR) of 0.5 for those with one operated hip, the actual burden on the non-operated hip remains uncertain and warrants further study. Additionally, our dataset does not include periprosthetic fractures, which may become more prevalent as the number of individuals with hip prostheses increases. These fractures fall under different diagnostic categories and were not analyzed in this study. It is therefore possible that the total burden of hip-related fractures is underestimated when periprosthetic complications are excluded. Future updates to national registries should aim to capture these outcomes in order to provide a more complete understanding of fracture risk dynamics in the aging population living with prosthetic hips.

The assumption of a fixed relative risk (RR = 0.5) for individuals with one hip replaced was derived from the NOREPOS study conducted in Norway. This estimate is supported by robust, long-term observational data and offers a reasonable approximation for modeling. However, we acknowledge that applying this assumption to the Romanian population may be an oversimplification, as it does not account for potential differences in surgical quality, postoperative care, or patient health profiles. While a full-range sensitivity analysis using RR values between 0.4 and 0.6 was not included in the current study, we recognize its value and will consider it in future iterations of the model. Notably, we did conduct a sensitivity analysis on the prevalence of prosthesis wearers, which demonstrated model stability even under ±20% variation. These findings lend confidence to the robustness of the main results, though further refinements are warranted.

**Figure 3:**
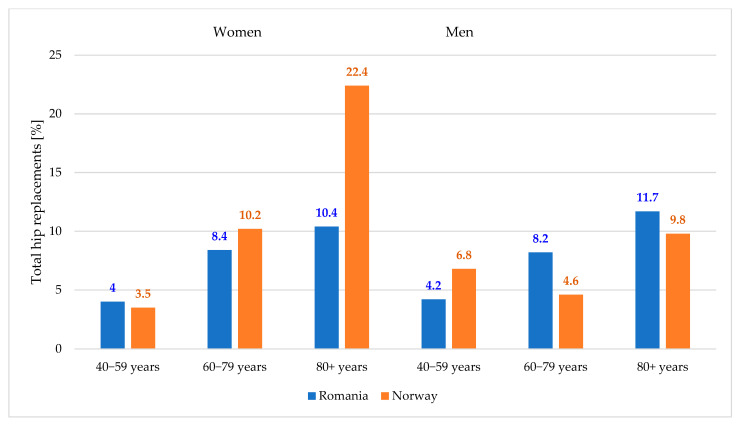
Comparison of the model results in Romania and Norway, indicating the prevalence of total hip replacements on the decrease in the share of hip fracture rates by gender and age groups.

In Figure 3, we compare the results provided by the model in terms of percentage prevalence of total hip replacements on the decrease in hip fracture rates in Romania and Norway. These are the only countries to which this model has been applied.

The results of our study are generally consistent with the analyses conducted in Norway by Kjeldgaard et al. [21]. The influence of total hip replacements on the decrease in fracture rates is approximately twice as large in Norway as in Romania for women over 80 years of age. This may be a consequence of the increased volume of THA interventions in women over men, and their longer life expectancy [29]. The other influences by gender and age group have comparable values, which demonstrates the consistency of the model.

In Romania, osteoarthritis is the main indication for hip replacement [30]. Sex- and age-standardized rates of THA have increased over time [31]. The way in which osteoarthritis influences the risk of hip fracture is not elucidated [32]. A high body mass index constitutes a strong risk for hip osteoarthritis [33,34], which in turn is associated with a higher risk of falling [35]. However, it is not clear how a THA procedure on one hip influences the risk of fracture in the controlateral non-operated hip [36]. In the absence of such information, it can be assumed that there is no influence and that the risk of hip fracture after THA is comparable to the population that has not undergone such interventions. We used the assumption that, compared with a person who did not undergo THA and has two intact hips, a person with one operated hip has RR=0.5 for a new hip fracture.

According to projections made by the National Institute of Statistics, the number of people aged 80 and over will increase by 50% between 2020 and 2060 [37]. Additionally, the burden on postmenopausal Romanian women is increasing [38]. As there is a sharp population aging phenomenon and that hip fracture rates continue to increase, additional interventions are needed to blunt this trend.

A strong point of the study is the mandatory reporting of THA interventions performed by orthopedics clinics to the Romanian Arthroplasty Register. Thus, complete national coverage, with accurate statistics after 2001, is obtained.

However, our study has some limitations. A first limitation is the use of data from national registers in Romania, which, compared with other countries, have a limited amount of information. At the same time, some information is not collected by the age groups. Specifically, the limitation is induced by the lack of accurate information regarding THA, prior to 2001, when RAR began systematic data collection at the national level. This may affect the accuracy of our calculations regarding the impact of THA on the hip fracture rate. Additionally, the absence of a national hip fracture database and the collection of data on the evolution of hip fractures over time may influence the calculations. As a result, the limitation comes from the low number and restricted periods of studies on the observed variation in hip fracture incidence in Romania. Another limitation of the study is due to the lack of information regarding the risk of hip fracture in patients who have already undergone THA. However, sensitivity analyses indicate the robustness of the calculations performed. Another limitation of this study is the use of secondary data from nationwide retrospective studies rather than direct access to primary patient-level datasets. Although these studies rely on DRG-based hospital reporting using ICD-10 coding (specifically code S72.0 for femoral neck fractures), potential misclassification or reporting inconsistencies cannot be entirely ruled out. However, the use of standardized coding across Romanian hospitals and the requirement for national data submission provide a high degree of reliability. Future research based on individual-level data or access to a centralized hip fracture registry would enable more detailed validation and stratification. An additional limitation of our model is the absence of adjustment for potential confounding factors such as diabetes, smoking, body mass index, use of osteoporosis medications, physical activity levels, and fall-prevention interventions. These factors are known to influence the risk of hip fractures and may have changed over time during the study period. While our model isolates the contribution of THA to the observed variation in fracture incidence, we acknowledge that the remaining variation is likely influenced by these and other unmeasured variables. The dark and bright segments in Figure 2 symbolically represent this division: the contribution of prosthesis use versus other factors not captured in our model. Future studies with access to individual-level clinical data are needed to quantify these effects more precisely.

Future research directions should include attempts to update the model with accurately calculated hip fracture risks for the subgroup of patients requiring total hip replacement, before and after THA. Conducting new studies at the national level, regarding the observed variation in hip fracture incidence over extended study periods, as well as diversifying data from national registries, will allow for new, much more detailed research, numerical simulation and automated data processing [39,40,41]. Another direction for research is to replicate the model in other countries to determine whether THA interventions, adjusted for age groups, make similar contributions to the variation in hip fracture rates.

## 5. Conclusions

Between 2008 and 2019, the age-standardized incidence of hip fractures increased in Romania, in both women and men. This is due to osteoarthritis, but it is not known how this influences the risk of hip fracture. In this study, we investigated whether there is a causal relationship between people living with hip prostheses and the risk of hip fracture.

The adapted version of the NOREPOS model applied in Romania to study the impact of total hip arthroplasty on the incidence of hip fractures between 2008 and 2019 showed that 10.16% of the decrease in the number of hip fractures could be explained by the people living with hip prostheses.

## Figures and Tables

**Table 1:** Annual number of hip fractures and age-standardized hip fracture incidence rates for men and women over 40 years old in the Romanian population and in the period 2008–2019.

			Women					Men		
Year	Number of Hip Fractures (NO)	Population (PO)	Crude Fracture Rate per 10,000 (r)	Standardized Rate Fracture Rate per 10,000 (sr)	95%CI	Number of Hip Fractures (NO)	Population (r)	Crude Rate Fracture Rate per 10,000 (PO)	Standardized Fracture Rate per 10,000 (sr)	95%CI
2008	7513	5,879,757	12.7	13.8	13.4–14.1	4266	5,537,747	7.7	7.6	7.3–7.7
2009	8138	5,830,977	13.9	14.9	14.4–15.2	4341	5,491,308	7.9	7.8	7.6–8.0
2010	8638	5,796,446	14.9	16.1	15.5–16.4	4635	5,548,789	8.3	8.3	8.1–8.5
2011	8653	5,768,005	15.0	16.2	15.7–16.5	4661	5,432,005	8.5	8.6	8.3–8.8
2012	9342	5,742,384	16.2	18.1	17.5–18.5	4781	5,407,876	8.8	8.9	8.6–9.1
2013	9797	5,721,101	17.1	19.1	18.5–19.5	4646	5,387,833	8.6	8.7	8.4–8.9
2014	10,004	5,699,711	17.5	19.5	18.9–19.9	4545	5,367,689	8.4	8.5	8.2–8.7
2015	10,362	5,672,982	18.2	20.3	19.6–20.7	4798	5,342,517	8.9	9.1	8.8–9.3
2016	10,784	5,640,532	19.1	21.9	21.2–22.4	4990	5,311,957	9.3	9.5	9.2–9.8
2017	11,076	5,608,023	19.7	22.6	21.8–23.1	5204	5,281,342	9.8	10.1	9.7–10.3
2018	11,512	5,575,173	20.6	23.7	22.9–24.2	5220	5,250,406	9.9	10.2	9.8–10.4
2019	11,948	5,544,784	21.5	24.6	23.8–25.2	5314	5,221,787	10.1	10.4	10.1–10.6

**Table 2:** The number and incidence rate of hip fractures in the Romanian population over 40 years, in 4-year age groups for women and men in 4-year intervals between 2008 and 2019.

		2008–2011			2012–2015			2016–2019	
Age	Number of Hip Fractures (NO)	Population (PO)	Fracture Rate per 10,000 Inhabitants (r)	Number of Hip Fractures (NO)	Population (PO)	Fracture Rate per 10,000 Inhabitants (r)	Number of Hip Fractures (NO)	Population (PO)	Fracture Rate per 10,000 Inhabitants (r)
Women									
40–44 years	157	1,852,704	0.84	121	1,817,759	0.66	107	1,780,533	0.60
45–49 years	266	1,713,053	1.55	273	1,680,742	1.62	258	1,646,322	1.56
50–54 years	643	1,680,468	3.82	609	1,648,772	3.69	735	1,615,006	4.55
55–59 years	1321	1,587,367	8.32	1293	1,557,427	8.30	1202	1,525,532	7.87
60–64 years	2599	1,510,074	17.21	2481	1,541,442	16.09	2464	1,509,874	16.32
65–69 years	4824	1,524,524	31.64	5062	1,484,351	34.10	4972	1,465,137	33.93
70–74 years	9388	1,047,383	89.63	9627	1,027,628	93.68	10,465	1,006,583	103.96
75–79 years	17,457	907,732	192.31	19,072	890,610	214.14	19,896	872,371	228.07
80–84 years	28,203	698,255	403.90	32,600	685,085	475.91	35,132	671,055	523.57
84+ years	40,215	349,127	1151.87	47,771	342,542	1394.60	54,006	335,527	1609.58
Men									
40–44 years	539	1,751,983	3.07	371	1,711,870	2.16	352	1,676,813	2.10
45–49 years	1055	1,619,924	6.51	921	1,582,835	5.81	839	1,550,420	5.41
50–54 years	1568	1,589,111	9.86	1232	1,552,727	7.93	1440	1,520,928	9.46
55–59 years	2268	1,501,071	15.11	2045	1,466,703	13.94	2048	1,436,666	14.35
60–64 years	3418	1,485,664	23.01	3132	1,451,649	21.57	3200	1,421,920	22.50
65–69 years	4759	1,441,645	33.01	4748	1,408,637	33.70	5209	1,379,783	37.75
70–74 years	7032	990,443	70.99	6868	967,766	70.96	12,808	947,947	135.11
75–79 years	10,549	858,384	122.89	10,914	838,730	130.12	11,234	821,554	136.74
80–84 years	15,959	660,029	241.79	17,373	645,177	269.27	17,999	631,964	284.81
84+ years	24,167	336,147	718.94	26,968	322,588	835.98	28,991	315,982	917.48

**Table 3:** Hip fracture incidence rates in 2008 and 2019 for women and men in three age groups over 40 years, observed and calculated in a scenario with population at risk with no hip prostheses.

Age	Number of Hip Fractures ^a^ (NO)	Number of Hip Prostheses ^b^ (NHP)	Population (PO)	Observed Fracture Rate per 10,000 Thousand Inhabitants (r)	Calculated Fracture Rate in Scenario per 10,000 Thousand Inhabitants ^c^ (rs)
Women 2008					
40–59 years	495	11,735	1,726,296	2.86	2.87
60–79 years	3952	27,825	1,277,671	30.93	31.27
80+ years	3066	1645	264,589	115.87	136.43
Women 2019					
40–59 years	417	26,173	1,627,948	2.56	2.58
60–79 years	4940	62,056	1,204,881	40.99	42.08
80+ years	6357	3669	225,553	254.77	256.66
Men 2008					
40–59 years	1004	10,415	1,625,882	6.17	6.18
60–79 years	2286	14,722	1,203,352	18.99	19.11
80+ years	976	637	249,198	39.16	39.21
Men 2019					
40–59 years	853	25,199	1,533,116	5.56	5.6
60–79 years	2607	35,621	1,134,694	22.97	23.00
80+ years	1758	1541	234,980	74.81	75.06

^a^ Number of hip fractures in the Romanian Arthroplasty Register [20]. ^b^ Number of prevailing hip prostheses in living individuals. ^c^ Fracture incidence per 10,000 thousand inhabitants is calculated in a scenario with no hip prostheses and subtracting 0.5 individuals from the number of populations at risk for each prevalent hip prostheses.

**Table 4:** The number of hip fractures attributable to the increased prevalence of hip prostheses from 2008 to 2019.

Gender	Age	Population in 2019 (PO)	Observed Number of Hip Fractures in 2019 (NO)	Fracture Incidence in Scenario 2008 (rs)	Expected Number of Hip Fractures in 2019 (NX)	Difference Between Observed and Expected Fractures in 2019 (NO)-(NX)	Number of Fractures Attributable to the Hip Protheses (NX^explained HP^)
Women	40–59	1,627,948	417	2.87	467	−50	2
	60–79	1,204,881	4940	31.27	3767	1173	98
	80+	225,553	6357	136.43	3404	2953	298
Men	40–59	1,533,116	853	6.18	948	−95	4
	60–79	1,134,694	2607	19.11	2168	439	36
	80+	234,980	1758	39.21	921	837	98

## Data Availability

The data used in this study can be requested from the corresponding author.
